# Pain Interference and Conscientiousness in Older Adults

**DOI:** 10.1093/geroni/igab046.3234

**Published:** 2021-12-17

**Authors:** Stephanie Judge, Kaitlyn Meyr, Suzanne Segerstrom

**Affiliations:** 1 University of Kentucky, Lexington, Kentucky, United States; 2 University of Kentucky, University of Kentucky, Kentucky, United States

## Abstract

Pain interference increases with age and occurs when pain interrupts daily activities. Individuals vary in their amount of interference at a given level of pain. Conscientiousness is a personality trait characterized by diligence, perseverance, and goal-directedness, and is associated with fewer unhealthy behaviors and better health, including less pain and fewer functional limitations. This study tested three hypotheses on the relationships between pain, Conscientiousness, and pain interference among community-dwelling older adults (N=210) in a longitudinal study. At semi-annual interviews, participants reported their pain and interference. Conscientiousness was measured at baseline and follow-up. Multilevel models tested the between- and within-person relationships among study variables. Greater pain predicted more interference (person: γ01=.541, SE=.042, p<.0001; visit: γ10 =.495, SE=.014, p<.0001) but higher Conscientiousness decreased interference (γ02=-.156, SE=.064, p<.025). There was an interaction between Conscientiousness and pain: At higher levels of pain, older adults higher in Conscientiousness experienced much less interference than their less conscientious peers (γ11=-.199, SE=.089, p<.025). Older age at baseline predicted a greater decrease in Conscientiousness over the study period (b=-0.013, t(91)=-2.07, p<.05). Conscientiousness reduces the negative impact of pain on daily function. This protective effect may reflect perseverance and commitment to valued activities, consistency with proactive health behaviors, or other attitudes and behaviors that reduce the likelihood of psychosocial sequelae of pain. Overall, the sample decreased in Conscientiousness over time; however, the direction and amount of change varied considerably. These results refine existing knowledge of personality in old age and implicate personality factors as a potential target for pain management.

